# Improved Preservation of Rat Small Intestine Transplantation Graft by Introduction of Mesenchymal Stem Cell-Secreted Fractions

**DOI:** 10.3389/ti.2024.11336

**Published:** 2024-06-19

**Authors:** Takumi Teratani, Yasuhiro Fujimoto, Yasunaru Sakuma, Naoya Kasahara, Masashi Maeda, Atsushi Miki, Alan Kawarai Lefor, Naohiro Sata, Joji Kitayama

**Affiliations:** ^1^ Division of Translational Research, Jichi Medical University, Tochigi, Japan; ^2^ Department of Surgery, Jichi Medical University, Tochigi, Japan; ^3^ Transplantation Surgery, Nagoya University Hospital, Nagoya, Aichi, Japan

**Keywords:** small intestine, preservation, mesenchymal stem cells, transplantation, graft survival

## Abstract

Segmental grafts from living donors have advantages over grafts from deceased donors when used for small intestine transplantation. However, storage time for small intestine grafts can be extremely short and optimal graft preservation conditions for short-term storage remain undetermined. Secreted factors from mesenchymal stem cells (MSCs) that allow direct activation of preserved small intestine grafts. Freshly excised Luc-Tg LEW rat tissues were incubated in preservation solutions containing MSC-conditioned medium (MSC-CM). Preserved Luc-Tg rat-derived grafts were then transplanted to wild-type recipients, after which survival, injury score, and tight junction protein expression were examined. Luminance for each graft was determined using *in vivo* imaging. The findings indicated that 30–100 and 3–10 kDa fractions of MSC-CM have superior activating effects for small intestine preservation. Expression of the tight-junction proteins claudin-3, and zonula occludens-1 preserved for 24 h in University of Wisconsin (UW) solution containing MSC-CM with 50–100 kDa, as shown by immunostaining, also indicated effectiveness. Reflecting the improved graft preservation, MSC-CM preloading of grafts increased survival rate from 0% to 87%. This is the first report of successful transplantation of small intestine grafts preserved for more than 24 h using a rodent model to evaluate graft preservation conditions that mimic clinical conditions.

## Graphical Abstract

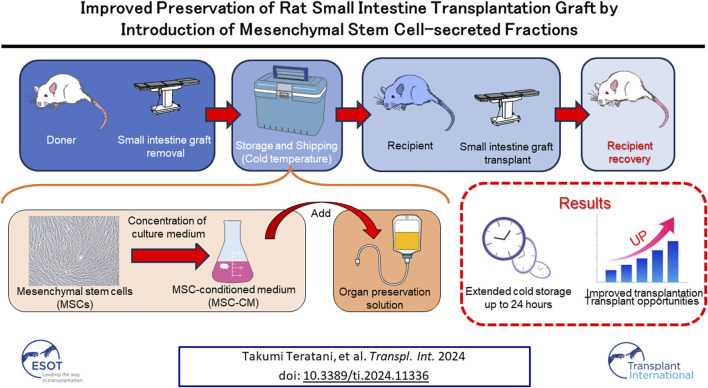

## Introduction

Recently, the management of intestinal failure has advanced with the establishment of the concept of an intestinal rehabilitation program. The resulting improved treatment results, especially for pediatric cases of intestinal failure, led to a reduction in number of annual intestinal transplants (ITx) worldwide to 149 in 2017 since reaching a peak of 270 per year in 2008 [1]. Nonetheless, ITx remains the ultimate alternative for patients requiring permanent parenteral nutrition. ITx using segmental grafts from living-related donors has recently been proposed to be advantageous [2]. Such grafts allow tissue matching, as well as shorter cold ischemic and operating times as compared with grafts from deceased donors [3]. Graft viability prior to implantation is a key factor in organ transplantation outcomes. When using grafts from brain dead donors, ischemia reperfusion injury and preservation damage affects graft quality, especially their barrier function [4]. During preservation, mucosal injury rapidly progresses to mucosal breakdown. Tissue injury worsens with reperfusion and further impairs the mucosal barrier, favoring bacterial translocation and sepsis [5]. In this context, successful preservation of graft viability during cold-ischemic storage is critical, with prevention of hypothermia-induced cellular swelling fundamental for successful organ preservation [6, 7]. The current clinical practice for intestinal preservation (IP) is based on an *in-situ* vascular flush with cold University of Wisconsin (UW) or Histidine-Tryptophan-Ketoglutarate solution, followed by cold static storage at 4°C [8, 9]. However, multiple studies have documented the inability of a variety of solutions, including UW solution, to maintain a clinically acceptable degree of morphological injury beyond 6–10 h of cold storage [10–12]. A breakthrough in the development of improved preservation solutions is important to address this problem.

Mesenchymal stem cells (MSCs) are multipotent and capable of differentiating into multiple lineages (osteogenic, chondrogenic, adipogenic, and neuronal) when cultured under defined *in vitro* conditions, rendering these cells useful for basic research and as a therapeutic cell source for clinical applications [13–15]. Recently, MSCs transplantation has been used to treat several human diseases as cell products authorized for commercialization by regions/countries [15–19]. These cells have broad utility with many therapeutic effects regarding organ injury and organ transplantation. Their effects on organ injury have been attributed to many secreted factors containing cytokines, which can inhibit inflammatory and immune responses [20–25]. There is also increasing evidence suggesting that the therapeutic potential of MSCs could be applied to difficulties encountered with ischemia reperfusion injury of the small intestine in experimental animal models, though application of MSCs to cold storage injury has not been adequately investigated [26–29].

In this study, we identified factors secreted from MSCs that allow direct activation of preserved small intestine grafts. This novel finding will help elucidate the precise molecular mechanisms of small intestine activation and will potentially be useful as an attractive source for preserved small intestine transplant therapy.

## Materials and Methods

### Animals

All animals were housed in a specific pathogen-free animal facility at Jichi Medical University under the following conditions: 50% ± 10% relative humidity, 12/12-h light-dark cycle, and a temperature of 24°C ± 2°C. Male wild-type Lewis (LEW) rats were purchased from Charles River (Breeding Laboratories, Kanagawa, Japan). LEW rats used in the experiments had a body weight between 230 and 310 g.

### Rat Adipose Tissue-Derived (rAT)-MSC Preparation and Culture

Wild-type LEW rat AT was sharply minced into pieces <3 mm, and rAT-MSCs isolation proceeded as described previously [30]. Isolated rAT-MSCs were seeded onto 100 mm tissue culture dishes (Nunc, Tokyo) and cultured with minimum essential medium (MEM)α supplemented with 10% fetal bovine serum. When the cells were 70%–80% confluent, they were harvested with 0.05% trypsin-EDTA (Invitrogen, Tokyo), replated at 2.0 × 10^4^ cells/cm [27], and cultured for 5 days. rAT-MSCs between the fifth and eighth passage were used for the experiments.

### Microarray Analysis

Clariom™ D Assay for Rat (Filgen, Tokyo, Japan) was used, according to the manufacturer’s instructions. Total RNA was extracted from undifferentiated rAT-MSCs and NRDFs. The process of hybridization and washing was performed using a Gene Expression Wash Pack (Agilent Technologies) and acetonitrile (Sigma, Tokyo, Japan). A DNA microarray scanner (Agilent Technologies) was used for array scanning. To ensure data reliability, weak signal spots were removed according to the manufacturer’s criteria. This resulted in a data matrix of 25,721-genes with no missing data.

### Preparation of Conditioned Medium

For analyses of secreted factors, rAT-MSCs were plated on 100 mm dish (using 30 dishes). Upon reaching confluence, samples were washed with phosphate-buffered saline (−) and incubated with serum-free MEMα medium. After 2 days, the supernatant was collected, centrifuged, filtered, and concentrated at 7000 × g using Amicon Ultra Centrifugal Filter Devices (Millipore, Tokyo, Japan; MW: 3 kDa, 10 kDa, 30 kDa, 50 kDa, and 100 kDa).

### Procurement of Small Intestine Segments

The firefly luciferase-expressing transgenic rat was established in our laboratory as described previously [31]. Small intestine segments from Luc-Tg LEW rats were removed at 8 weeks of age, divided into 10 mm segments after heparinization (300 U/animal), and washed with Hank’s Balanced Salt Solution (Invitrogen, Tokyo, Japan).

### Correlativity of Photon Intensity and Cell Viability

IVIS and trypan blue staining were used to confirm the correlation of photon intensity and cell viability. Muscularis and mucosa layers from Luc-Tg LEW rat small intestine were trypsinized to the cellular level. After preservation with normal saline at normothermic conditions, photon intensity and subsequently cell viability rates were evaluated by trypan blue staining on the same specimens.

### Assessment of Small Intestine Tissue Viability in Preservation Solutions

Freshly isolated Luc-Tg LEW rat small intestine segments were plated in 12 wells tissue culture plates (1 segment/well: *n* = 4/each), and stored in UW preservation solution (Astellas Pharma Inc., Tokyo, Japan) at 4°C for 24 h. Detection was performed by addition of 22 μL (2.29 mg/ml) of luciferase-based reagent (D-luciferin: Wako, Tokyo, Japan). The *in vivo* imaging system (IVIS; Xenogen, Allameda, CA, United States) was used for the analysis of luciferase gene expression activity. In this system, a non-invasive charged-couple device camera is used to detect bioluminescence emitted from D-luciferin, which reacts with firefly luciferase in living animals and cells.

### Immunohistochemical Analysis of Preserved Small Intestine Segment

Small intestine samples were stored for 24 h at 4°C, fixed in 10% formalin, and embedded in paraffin. Histological analysis of small intestine segments was conducted on serial tissue sections stained with hematoxylin and eosin (H&E) for conventional morphological evaluation and with anti-ZO1 (Hycult Biotech, Uden, Netherlands), anti-claudin-3 (Santa Cruz Biotechnology, CA, United States), and anti-myeloperoxidase (MPO) antibodies (Hycult Biotech) for protein detection. Rhodamine- or fluorescein isothiocyanate (FITC)-conjugated secondary antibodies were applied for 30 min. Nuclei were stained using 4′,6-diamidino-2-phenylindole (DAPI).

### Macroscopic and Microscopic Scoring for Small Intestine Injury

Both macroscopic and microscopic scoring systems were used for analysis of tissue damage. Subsequent to resection, the small intestine was immediately cut into 10-pieces of equal length and rapidly transferred to Petri dishes containing cold buffer on ice. Macroscopic changes in each piece were documented photographically. Histological grading of injury of formaldehyde-fixed intestine specimens, counterstained with H&E, was performed by 2-independent blinded examiners using the Park/Chiu classification [32].

### Heterotopic Transplantation of Preserved Partial Small Intestine Grafts With Double Enterostomy in Syngeneic Wild-Type Rats

Luc-Tg LEW rat derived small intestine grafts (10 cm) were transplanted into wild-type LEW rats, as described previously [3, 13]. In brief, a transverse incision was made on the donor abdomen, and the middle and right colonic vessels were ligated and divided. The distal end of the graft intestine was cut at the ileum end and the proximal end was subsequently cut 10 cm from the distal end. After venous injection of heparin, the aorta was ligated above the origin of the superior mesenteric artery, and the intestinal lumen flushed with an adequate amount of saline.

The aorta including the superior mesenteric artery was then divided. The portal vein was divided, the graft removed *en bloc* and the vessels flushed. The graft was stored immediately in UW solution or UW + MSC-CM at 4°C. After 24 h of cold storage, the graft was placed in the recipient abdomen, the recipient’s aorta was partially side-clamped, and the graft aortic conduit was anastomosed to the infrarenal abdominal aorta in an end-to-side fashion. Venous outflow was restored by end-to-side anastomosis of the portal vein to the infrarenal vena cava. Both ends of the small intestine graft were exteriorized as stomas. The incision was closed with interrupted sutures.

This rodent model was designed to investigate the status of the preserved graft and is not a sublethal model due to heterotopic partial transplantation. In a preliminary study, all recipients survived with saline transfusion at the end of the transplant procedure. Therefore, the transplant procedures were performed without transfusing saline to make this model sublethal. On postoperative days (PODs) 1, 3, and 7, D-luciferin (150 mg/kg body weight) was injected into the penile vein of each rat, and the rat anesthetized with isoflurane (Abbott Japan Co., Ltd.), to detect photons emitted from the small intestine graft. Graft luminescence was evaluated by the IVIS and quantified with the IVIS Living Image software package.

### Statistical Analysis

Data are represented as means ± standard error of the mean (SEM). Pearson correlations were performed to determine the association between cell viability and relative photon intensity. To compare the mean values of relative photon intensity obtained from the preserved small intestine segment, repeated measures single-factor ANOVA followed by Tukey’s multiple comparisons test was performed. For the survival study, Kaplan-Meier analysis and the log-rank test were performed. Mean values of photon intensity to compare the two groups were analyzed using a 2-tailed Student’s *t-*test. Mann-Whitney’s U-test was used to compare injury scores between the groups. A *p*-value < 0.05 was considered significant. All statistical analyses were performed using the GraphPad Prism software.

## Results

### Luminescence Technology to Assess Viability of Preserved Small Intestine *In Vitro*

**FIGURE 1:**
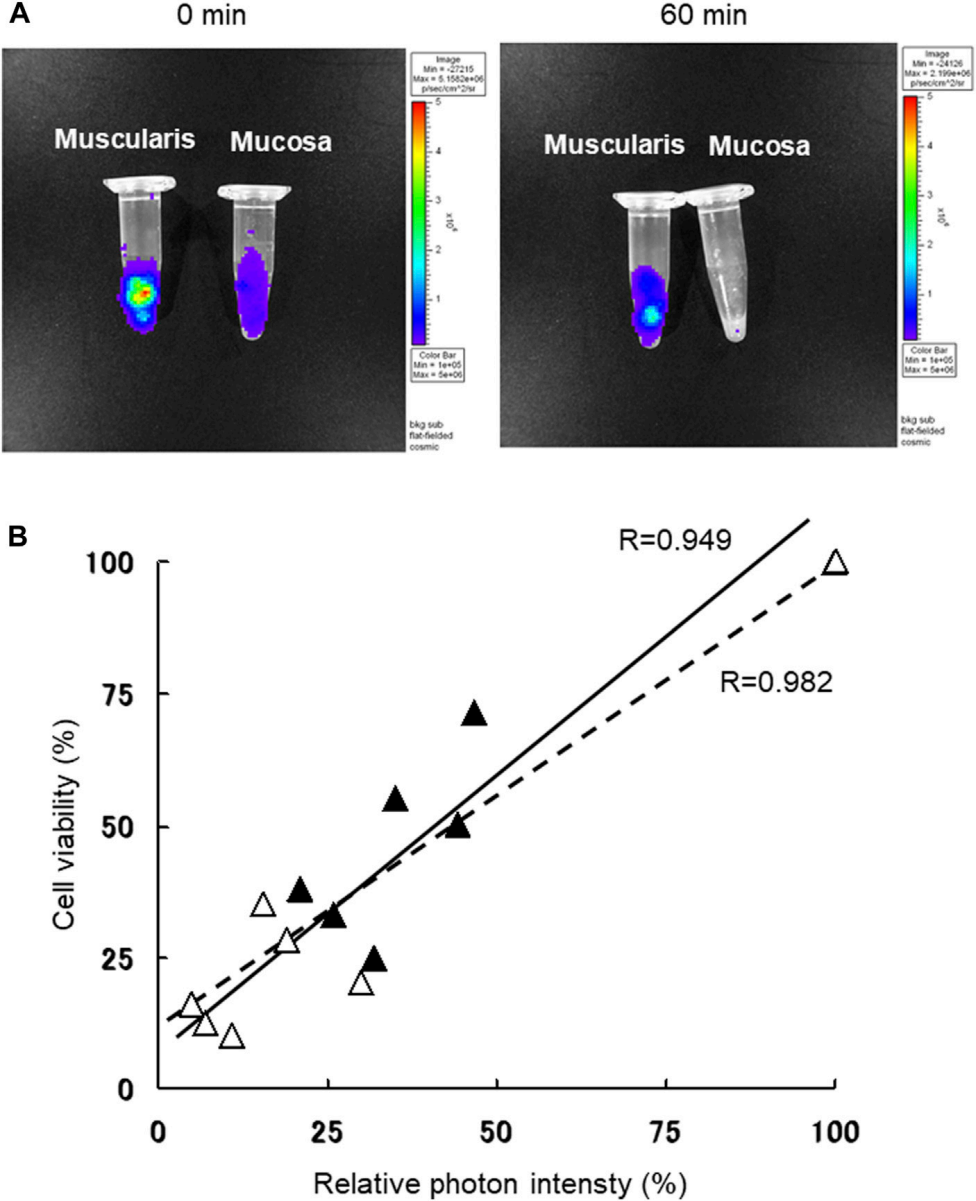
Small intestine muscularis and mucosal viability were determined following trypan blue staining, based on bioluminescence with use of *in vivo* imaging system (IVIS). **(A)** Luciferase transgenic (Luc-Tg) LEW rat-derived small intestine tissues were separated into muscularis and mucosa before normothermic normal saline preservation. Using the IVIS, the image on the left was acquired at the starting time and on the right image after 60 min of preservation. **(B)** Photon count of Luc-Tg LEW rat trypsinized small intestine tissues was determined using the IVIS, followed by evaluation of cell viability with trypan blue staining. Black triangles and straight lines show data for muscularis, and white triangles and broken lines show data for mucosa under the same conditions shown in **(A)**.

Using a stereomicroscope, small intestinal tissue from Luc-Tg LEW rats was strictly separated into muscularis and mucosal layers. The photon intensity from the muscularis and mucosal layers decreased as preservation time extended (Figure 1A). The viability of samples preserved in normal saline was lower in the mucosal layer than in the muscularis layer after 1-h. The photon count for trypsinized tissue samples preserved with normal saline under normothermic conditions was strongly correlated with cell viability, evaluated following trypan blue staining (Figure 1B). Both the muscularis and mucosal layers showed similar correlativity. These results demonstrate the validity of using a whole section of small intestine wall to accurately evaluate the state of preservation by measuring the photon intensity using the IVIS.

### Analysis of Preserved Small Intestine Activation Factors From rAT-MSC-Secreted Fractions

**FIGURE 2:**
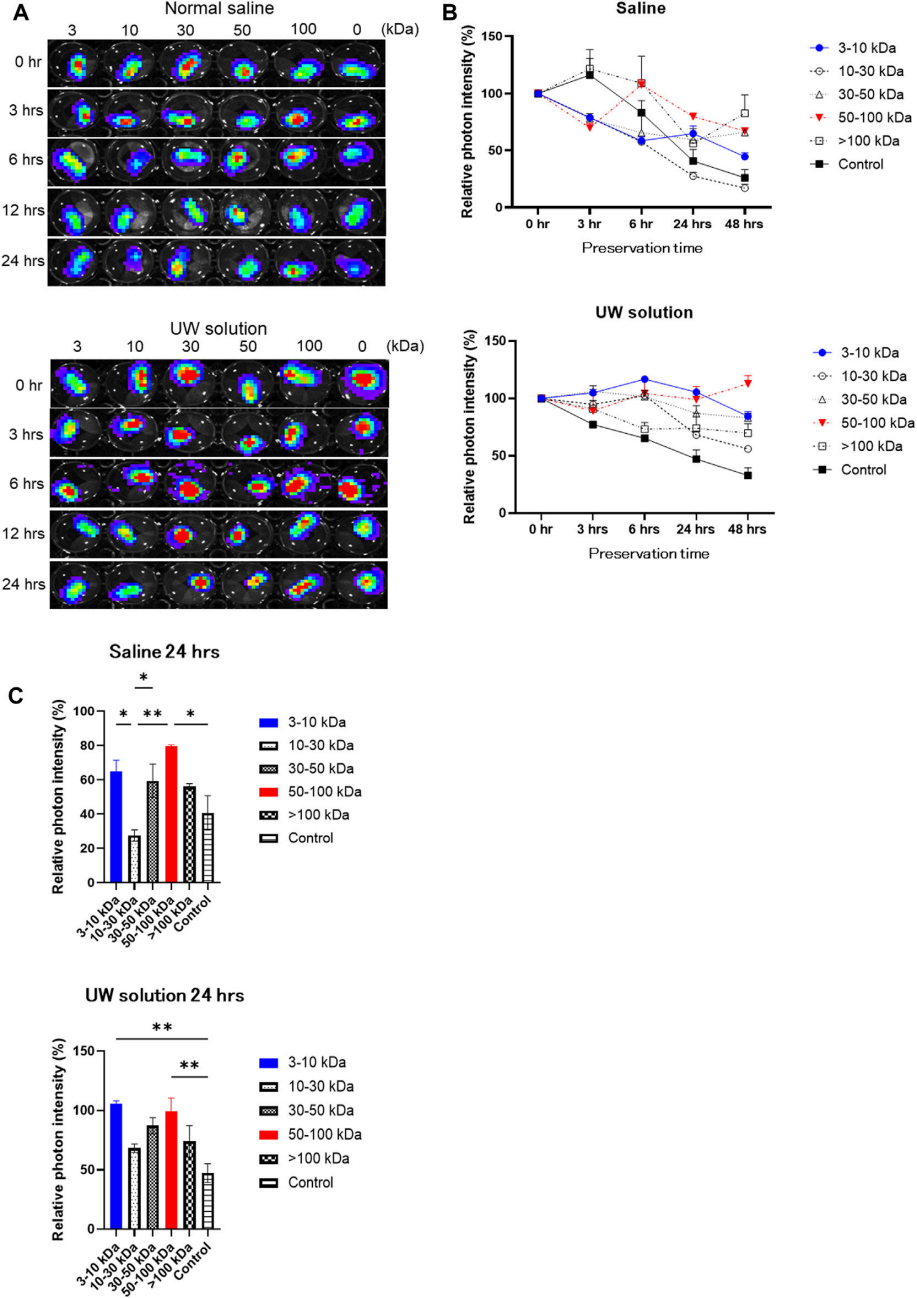
Comparison of changes in luminescence intensity of small intestine segments in organ preservation solution following addition of each fraction of mesenchymal stem cell-conditioned (MSC) medium. **(A)** Representative time-lapse photographs of Luc-Tg LEW rat-derived small intestine segments in preservation solution treated with each fraction of MSC-conditioned medium. Shown from the left column on the plate; >100 kDa, 50–100 kDa, 30–50 kDa, 10–30 kDa, 3–10 kDa, and 0 kDa (control) fractions. **(B,C)** Relative photon intensity of small intestine segments determined using *in vivo* imaging system to assess viability. Samples were immersed in organ preservation solution at 4°C. Data shown are representative of 3 independent experiments.

For the result of DNA microarray analysis, rAT-MSC-CM contains 29 growth factors that affect the viability of cold-preserved small intestine (Table 1). Fractions derived from rAT-MSC-CM were evaluated to determine which were involved in activation of the preserved small intestine (Figure 2). During the experiment, the preservation solution was not refreshed. The photon intensity from the group receiving each fraction of conditioned medium changed over time at 4°C (Figure 2A). The photon intensity was quantified using color images. By comparison with controls, fractions were classified into 2 groups in terms of their effects on preserved Luc-Tg LEW rat small intestine grafts as follows: activated group (30–100 and 3–10 kDa) and less activated group (0–3 and 10–30 kDa) (Figure 2B). The activation of preserved small intestine grafts gradually declined in photon intensity from the peak at 24 h (Figure 2C), then fell below the detection limit within 4–5 days (data not shown). These results suggest that the 30–100 and 3–10 kDa fractions secreted by rAT-MSCs were superior in their activation of preserved small intestine grafts.

**TABLE 1:** Upregulation of cytokine genes in rAT-MSCs as compared with NRDFs (*p* < 0.001).

Name	rAT-MSCs	NRDFs	Ratio
FGF1	1,004.07	254.31	3.95
FGF2	5,011.34	633.14	7.92
FGF4	36.65	24.57	1.49
FGF5	693.31	6.31	109.96
FGF7	143.98	6.95	20.73
FGF16	45.63	7.61	6.00
FGF18	232.53	23.01	10.11
FGF22	3,462.66	630.26	5.49
FGF23	27.21	6.57	4.14
HGF	127.01	6.76	18.78
VEGF	1,157.93	845.47	1.37
VEGFC	14,993.36	7.69	1949.14
VEGFFB	18,723.8	1,126.92	16.62
VEGFB	790.95	55.6	14.23
EGF	45.78	7.79	5.87
NGF	199.14	76.37	2.61
IGF1	13.26	5.17	2.56
IGF2	1,315.06	39.85	33.00
TGFB1	1,245.00	71.27	17.47
TGFB3	37.00	19.64	1.88
HDGFL1	73.58	30.78	2.39
PGF	368.80	221.08	1.67
PDGFC	5,713.71	2,181.63	2.62
PDGFD	26.79	6.94	3.86
BCGF	19.41	7.04	2.76
TBRG1	283.64	126.22	2.25
LTBP1	2,420.02	712.98	3.34
HDGFRP3	2,686.30	14.08	190.79
CTGF	13,349.88	5,469.09	2.44

### Histological Analysis of Preserved Small Intestine Segments

**FIGURE 3:**
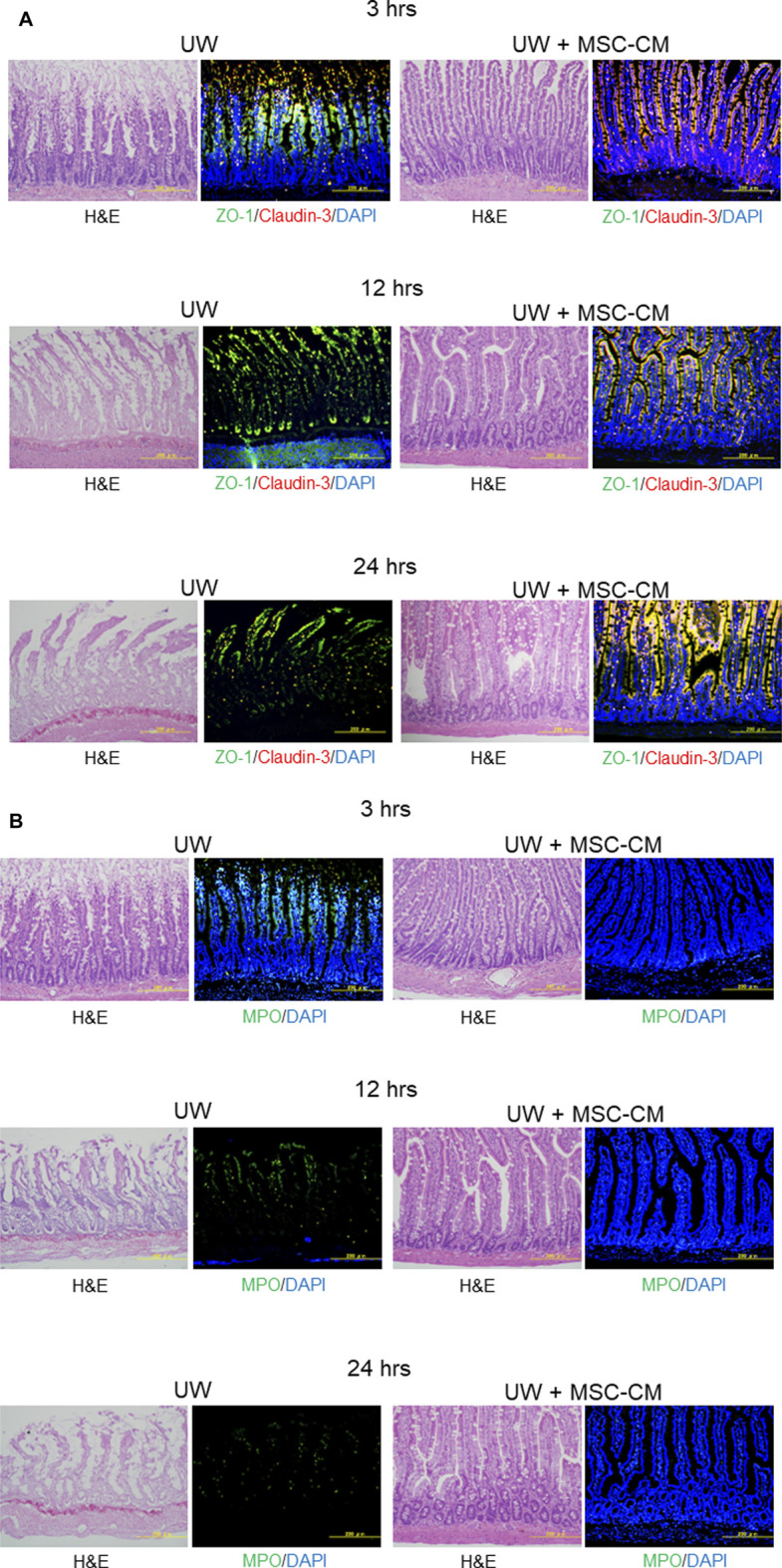
Assessment of tight-junction structure and oxidative stress. **(A)** Microphotographs of preserved small intestine segments. Left two columns show hematoxylin and eosin staining and immunofluorescence images of segments preserved in University of Wisconsin (UW) solution showing expression of ZO-1 (green), claudin-3 (red), and their colocalization (yellow), while right two columns show segments preserved in UW + MSC-CM at each time point. Nuclei were stained blue with DAPI. **(B)** Evaluation of oxidative stress marker MPO (green) in the same samples. The samples used were serial sections.

Claudin-3 and ZO-1 were found to be colocalized in epithelial villi on the surface of enterocytes in normal intestine specimens. After 3 h, the segments in UW solution alone tended to be delocalized along the intercellular membrane due to a decrease in claudin-3 expression, but colocalization was maintained in the region closer to the top of the villi (Figure 3A; Table 2). After 12 h of preservation, the expression of claudin-3 was remarkably decreased and ZO-1 expression was also decreased, in a discontinuous pattern in UW solution alone. After 24 h of cold preservation, most of the villus structure was destroyed in segments of intestine in the UW solution alone group. The expression of Claudin-3 almost disappeared, while a quantity of ZO-1 expression at the top of the villi was maintained. Only minimal change was observed in segments preserved in the UW + MSC-CM solution with respect to histological architecture, expression and colocalization of two proteins at all time points.

**TABLE 2:** Immunohistochemical results of rat small intestine grafts preserved in mesenchymal stem cell-conditioned medium.

MSC-CM (kDa)	Preservation time (hr)	Claudin-3	ZO-1	MPO	DAPI
0<Factor<3	3	+	+	+	+
	12	−	+	−	−
	24	−	+/−	−	−
3<Factor<10	3	+	+	-	+
	12	+	+	+	+
	24	+	+	+	+
10<Factor<30	3	+	+	+	+
	12	+/−	+	+	+
	24	−	+/−	+	+
30<Factor<50	3	+	+	−	+
	12	+	+	−	+
	24	+/−	+	+	+
50<Factor<100	3	+	+	−	+
	12	+	+	−	+
	24	+	+	−	+
100<Factor	3	+	+	+/−	+
	12	+	+	+	+
	24	+/−	+	+	+

Immunohistochemical staining of MPO expression was also performed to evaluate oxidative stress in preserved small intestine segments. The MPO expression was evidently reduced in the UW + MSC-CM group compared to the UW solution alone group at all time points (Figure 3B; Table 2).

### Preserved Small Intestine Graft Transplantation

**FIGURE 4:**
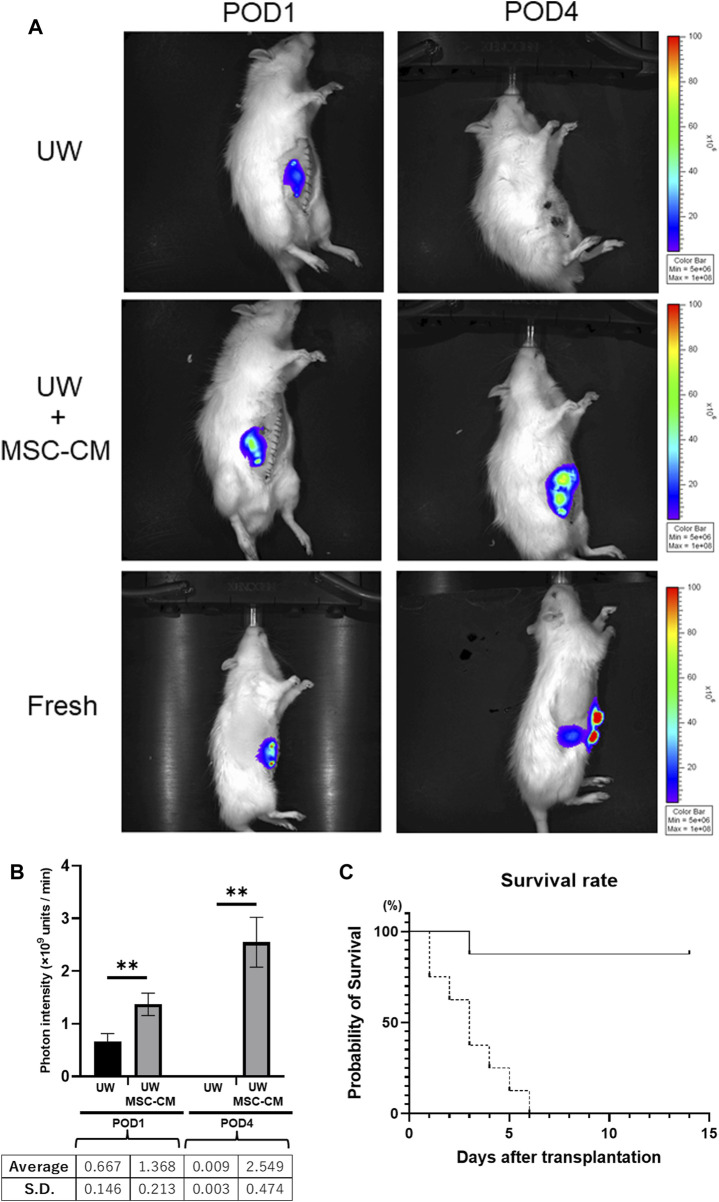
Time course of preserved Luc-Tg derived intestine grafts in recipient. **(A)** Changes in luciferase-derived photons following transplantation of small intestine. Representative image from each group on post-operative day (POD)1 and POD4. The Fresh group included transplanted grafts without preservation. **(B)** Graph showing luminescent photon level up to 4 days following transplantation. Black bar represents University of Wisconsin (UW) alone group and white bar UW + MSC-CM group. **p < 0.05. **(C)** Survival rate of rats following transplantation. Solid line: UW + MSC-CM group (*n* = 8), dashed line: UW alone group (*n* = 8).

Luc-Tg LEW rat-derived small intestine grafts were transplanted into wild-type LEW rats after 24 h of cold preservation. The condition of the transplanted small intestine graft was evaluated daily based on the presence of relative photons using the IVIS. In the control group without MSC-CM, photon intensity showed significant attenuation over time, whereas that was increased in the group containing MSC-CM (>50 kDa fraction), similar to the increase seen in non-preserved small intestine grafts (Figure 4A). The small intestine graft preserved for 24 h with either UW alone or UW + MSC-CM did not show any changes at the pre-transplant stage. Furthermore, the photon intensity rate from transplanted small intestine grafts preserved in UW solution alone decreased more rapidly than those in UW + MSC-CM [Figure 4B; UW: 0.6 ± 0.16 units/min (POD1), 0.009 ± 0.003 units/min (POD4); UW + MSC-CM: 1.38 ± 0.21 units/min (POD1), 2.55 ± 0.47 units/min (POD4)]. After POD14, the UW solution alone and UW + MSC-CM groups had photon intensities very similar to that of fresh small intestine transplanted grafts (Figure 4B).

Saline-transfused recipient rat survival was 100%; however, control rats without saline transfusion [the UW solution alone group (*n* = 8)], had 100% mortality by POD6 (Figure 4C). In the UW solution alone group, bleeding into the peritoneal cavity was confirmed at autopsy. In contrast, infusion of saline was unnecessary in UW + MSC-CM rats for survival.

### Gross Appearance of Transplanted Cold-Preserved Small Intestine Rat Grafts

**FIGURE 5:**
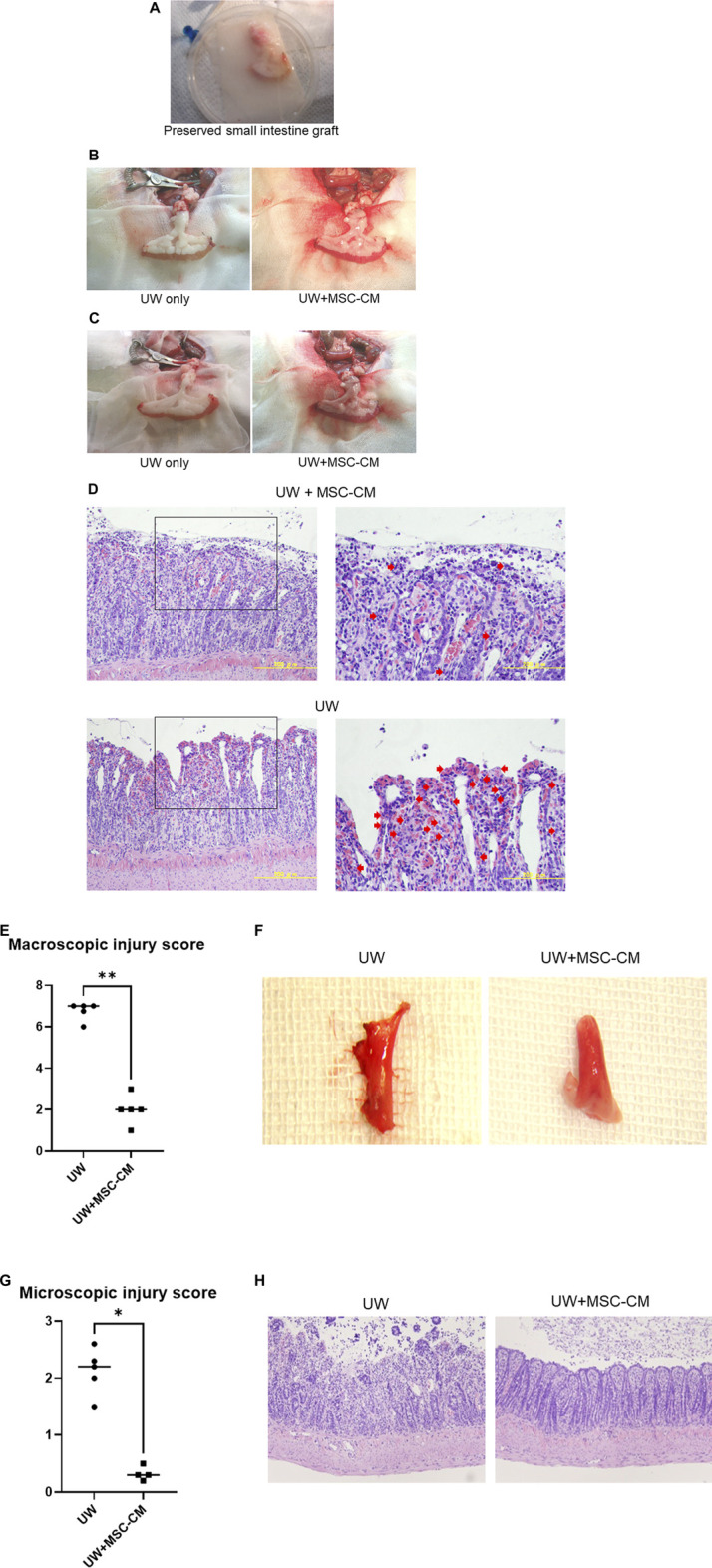
Gross appearance of preserved small intestine grafts before and after reperfusion. **(A)** Preserved small intestine graft. **(B)** Before reperfusion (left: University of Wisconsin (UW) alone, right: UW + mesenchymal stem cell-conditioned medium (MSCX-CM). **(C)** Following reperfusion. Left: UW alone, right: UW + MSC-CM. **(D)** Representative section of small intestine graft at 24 h after transplantation. Red arrows indicate typical apoptotic cells. **(E–H)** Analysis of small intestine preservation and reperfusion injury levels in heterotopic partial transplantation model. **(E)** Macroscopic injury score. **(F)** Morphology of whole small intestine grafts. **(G)** Microscopic injury score. **(H)** Hematoxylin and eosin (H&E)-stained sections of small intestine grafts under various conditions. *p < 0.05 **p < 0.01.

Approximately 10 cm of ileal graft was removed under general anesthesia and kept at 4°C for 24 h in UW solution alone or UW + MSC-CM solution (Figure 5A). The small intestine graft preserved for 24 h with either UW alone or UW + MSC-CM did not show any changes at the pre-transplant stage. After 24 h storage, preserved grafts were transplanted into the peritoneal cavities of recipient rats and reperfused. Grafts preserved in UW solution alone were found to be edematous and congested with blood oozing from the mucosa, while those preserved in UW + MSC-CM did not have those findings (Figures 5B, C). Furthermore, grafts preserved in UW solution alone harvested 24 h after transplantation had marked congestion and bleeding in microscopic sections evaluated with hematoxylin-eosin stain. Karyopyknosis and deciduation of intestinal epithelial cells were also observed as typical apoptosis (Figure 5D). However, grafts preserved in UW + MSC-CM solution had mild congestion and apoptosis.

After 90 min of reperfusion, small intestine graft conditions were obviously different between those preserved in UW solution alone and those in UW + MSC-CM, with the macroscopic score significantly lower in the latter (Figures 5E, F). Using the same samples, small intestine histopathology was examined and the microscopic score values were consistent with the macroscopic results (Figures 5G, H). These results suggest that UW + MSC-CM solution is beneficial for long-term storage of small intestine grafts under ischemic conditions.

## Discussion

The results of the present study demonstrate for the first time that isogeneic adipose derived MSC-CM supplementation of UW solution has a protective effect on intestinal grafts against 24 h extended cold static preservation in a rodent heterotopic ITx model. In addition, particular fractions of MSC-CM, including the 30–100 and 3–10 kDa fractions, were found to contain trophic factors that allow maintenance of tissue ATP content after 24 h as compared to the conditions at the initiation of storage. This method does not require complex equipment to control oxygenation or temperature, and has practical benefits, given that cold static preservation is standard practice for intestinal preservation.

Although MSCs are used for treatment of several different diseases and have recently been commercialized as therapeutic products, an urgent issue to overcome is cell supply to meet sudden demand in acute clinical situations [33, 34]. For a single treatment in clinical settings, hundreds of millions of MSCs are generally needed to attain an adequate therapeutic effect. Since several weeks are required to increase the number of cells grown in normal culture conditions, investigators have found it difficult to apply MSC-based cell therapy, especially for acute diseases. On the other hand, it has been shown that the effect of MSCs is due at least in part to paracrine factors [35]. We conducted analysis of DNA microarray and Protein-chip array to explore secretory factors of AT-MSCs (preparation of manuscript). Administration of MSC-CM in acute organ injury models has been reported to be as effective as administration of MSCs in some cases [36, 37]. MSC-CM is known to contain various cytokines, such as growth, anti-inflammatory, and anti-apoptotic factors, which regulate a large variety of physiological processes. Thus, cell-free therapy methods such as use of MSC-CM have been attracting attention due to some crucial advantages over stem-cell based applications.

Use of UW solution to flush the vasculature and store donor organs during retrieval has been the standard method employed for abdominal organ preservation since 1987 [24]. Despite the effectiveness of cold static preservation with UW solution for other intra-abdominal organs, that for small intestine preservation is somewhat limited. The maximum “safe” storage time for small intestines is brief (6–12 h) and graft quality is often compromised even with short periods of ischemia [22, 38]. With cold preservation, hypothermia elicits protective effects by delaying hypoxia-induced ATP decline and slowing down subsequent injury [39]. A previous report showed that AMP nucleosidase gene knockout in *Escherichia coli* elevates intracellular ATP levels and increases cold tolerance [40]. In addition, elevated intracellular ATP levels by deactivation of AMP deaminase was demonstrated with maintenance of decreased adenylate kinase activity in specific hibernating mammals [41]. Thus, elevation of intracellular ATP levels is critically important for cell survival at low temperatures. During cold static organ preservation, minimum cellular metabolism factors related to cell survival, including ATP synthesis and amino acid metabolism, are profoundly suppressed, which prevents consumption of essential substrates. Therefore, organs stored for a comparatively long time at low temperature cannot be revived due to irreversible changes in the energy synthesis components needed for cell survival.

Maintenance of high-energy phosphorylated compounds such as ATP, the levels of which are inversely proportional to preservation time, has been shown to be correlated with minimal changes in cell structure and function incurred during cold storage [11]. The Na^+^/K^+^ transporter maintains the respective gradients of such ions, relying on use of ATP as its energy source. An essential factor for successful intestinal graft transplantation under cold ischemic conditions is the ability of the preservation solution to maintain ATP levels [12]. UW preservation solution maintains ATP synthesis in the graft tissues and ameliorates the effects of cold ischemia up to 12 h. We previously presented an assay used to assess the viability of Luc-Tg LEW rat organs and tissues [42–47], and that was used in the present study to confirm that small intestine segments preserved for 24 h in 3–10 kDa and 50–100 kDa fractions of MSC-CM showed nearly equivalent photon intensity levels, which reflects tissue ATP quantity, as compared to that at the initiation of cold ischemia. The present findings also showed that grafts after preservation in UW solution alone for 24 h had blood oozing from the entire surface of the tissue upon resumption of blood flow, while grafts preserved in UW + MSC-CM solution did not. These results are consistent with histological analysis findings showing destruction of the intestinal configuration including microvasculature in the UW solution alone group. HGF is known to suppress ischemia-reperfusion injury associated with organ transplantation, particularly apoptosis [48–50]. Findings obtained with cytokine arrays have shown that AT-MSCs secrete HGF at levels approximately 10-fold greater as compared to BM-MSCs [17]. Furthermore, MSC transplantation is known to increase graft survival rates and suppress rejection reactions [51–53]. Thus, when using MSCs for this purpose, those derived from autologous tissues are best so as to evade rejection reactions. These findings directly reflect the results showing significantly improved recipient survival up to 87% using grafts preserved with MSC-CM as compared to recipients that received grafts preserved without MSC-CM, which died within 6 days following transplantation.

Major obstacles to development of reliable and safe ITx methods are largely related to bacterial infection. Cytokines, interferon-γ, and tumor necrosis factor (TNF)-α directly influence tight junction function, and also modulate both membrane microdomain localization of tight junction proteins and lipid composition of tight junctions, resulting in bacterial infection and inflammation [54]. HGF is known to suppress ischemia-reperfusion injury associated with organ transplantation, particularly apoptosis [48–50]. Through cytokine arrays, it has been found that AT-MSCs secrete approximately 10 times more HGF than BM-MSCs [17]. Furthermore, MSC transplantation is known to increase graft survival rates and suppress rejection reactions [51–53]. When using MSCs for this purpose, it is desirable that they are derived from autologous tissues to evade the MSC’s own rejection reactions. Therefore, to maintain the efficiency of small intestine grafts at an optimal level, it is important to protect grafts against tight junction destruction during cold preservation and subsequent reperfusion. The present findings show that UW solution containing MSC-CM inhibits tight junction breakdown and maintains cell structure in the small intestine under cold preservation for 24 h (Figure 3). Furthermore, grafts preserved under these conditions were found to be transplantable into recipient rats (Figure 5). Mitochondrial DNA, which is released from dead cells and can induce an inflammatory response, has been shown to contribute to intestinal ischemia reperfusion injury and exacerbate the acute proinflammatory process by enhancing production of proinflammatory cytokines including TNF-α [55]. It is thus possible that reduction of tissue damage during preservation caused by addition of MSC-CM leads to a lower level of mitochondrial DNA circulation after reperfusion and suppresses the proinflammatory process, resulting in improved survival of the recipient. As noted above, it is best to use MSCs derived from autologous tissues so as to evade their rejection reactions.

Methods to expand the donor organ pool have been developed over the previous decade. Included in the marginal donor group are donated organs recovered after cardiac death, formally known as non-heart-beating donation [56]. As compared with organs transplanted after brain death, the function of organs obtained from donors after cardiac death is poor, though there is potential for significant improvement. Notably, the present results show that the 50–100 kDa fraction of MSC-CM allows grafts to attain a greater level of photon intensity after cold preservation extended to 48 h as compared to that at the beginning, which indicates maintenance of ATP production by tissue metabolism. Extrapolation of this finding suggests that use of MSC-CM as an adjunct to UW solution could contribute to improve the function of grafts obtained as donations after cardiac death that have severe ischemic injuries by *ex vivo* recovery of ATP production.

The present study has some important limitations. Since the animal model of ITx was produced using inbred syngeneic animals without rejection, assessment of the immunomodulatory effect of MSC-CM was not feasible. In addition, the present was a partial heterotopic transplantation model with a so-called Thiry-Vella loop and the native bowel in the recipient animal remained intact. MSCs are known to secrete exosomes [57–59]. Chai et al. reported the presence of exosomes derived from MSCs in the 100 kDa–1,000 kDa fraction, which have been reported to alleviate ischemia-reperfusion injury [57]. It is suggested that our fractions above 100 kDa contain not only cytokines but also exosomes. In this study, examinations of the effects of isolated exosomes were not performed, thus it will be necessary to examine the interaction between cytokines and exosomes in a future study, while the ability to evaluate the efficacy of MSC-CM on graft intestine motility and digestive absorption function was also limited.

In conclusion, small intestine grafts maintained in cold storage with MSC-CM supplementation for 24 h were successfully transplanted in the present rat model under conditions similar to those used in clinical practice and the recipient survival rate showed dramatic improvement. Additional investigations are needed to clarify the underlying mechanisms and identify the most important factors contributing to the observed benefits when using MSC-CM so as to expand use of this approach to clinically relevant applications. As for the translational impact, it is suggested that the present results may have significant influence on discovery of pathways related to extending the preservation time of various transplant organs as well as research related to drug development.

## Data Availability

The original contributions presented in the study are included in the article/supplementary material, further inquiries can be directed to the corresponding author.

## Abbreviations

rAT-MSCs, rat adipose tissue-derived mesenchymal stem cells; CM, conditioned medium; Luc, luciferase; Tg, transgenic; LEW, Lewis; NRDFs, neonatal rat derived fibroblasts; ITx, intestinal transplants; UW, University of Wisconsin.
